# *In vitro* protein binding of liraglutide in human plasma determined by reiterated stepwise equilibrium dialysis

**DOI:** 10.1002/jps.23648

**Published:** 2013-07-12

**Authors:** Anne Plum, Lisbeth Bjerring Jensen, Jesper Bøggild Kristensen

**Affiliations:** 1 Department of Diabetes Pharmacology PK/PD, Novo Nordisk A/SMåløv DK-2760, Denmark; 2 Department of DMPK, Cell and Antibody Analysis, Novo Nordisk A/SMåløv DK-2760, Denmark; 3 Chemistry and Isotope Laboratory, Novo Nordisk A/SMåløv DK-2760, Denmark

**Keywords:** liraglutide, type 2 diabetes mellitus, protein binding, plasma proteins, human serum albumin, *in vitro* method, equilibrium dialysis, insulin detemir, acylated peptides

## Abstract

Liraglutide is a human glucagon-like peptide-1 (GLP-1) analogue approved for the treatment of type 2 diabetes. It is based on human GLP-1 with the addition of a 16-carbon fatty acid, which facilitates binding to plasma proteins, thus prolonging the elimination half-life and allowing once-daily administration. It has not been possible to quantify liraglutide protein binding by ultrafiltration (the usual method of choice), as the lipophilic molecule becomes trapped in the filter membrane. Therefore, the aim of this study was to develop a methodology that could determine the extent of liraglutide binding to plasma proteins *in vitro*. We report here the details of a novel reiterated stepwise equilibrium dialysis assay that has successfully been used to quantify liraglutide plasma protein binding. The assay allowed quantification of liraglutide binding to proteins in purified plasma protein solutions and human plasma samples and was effective at plasma dilutions as low as 5%. At a clinically relevant liraglutide concentration (10^4^ pM), greater than 98.9% of liraglutide was bound to protein. Specific binding to human serum albumin and α1-acid glycoprotein was 99.4% and 99.3%, respectively. The novel methodology described herein could have an application in the quantification of plasma protein binding of other highly lipophilic drug molecules.

## INTRODUCTION

Glucagon-like peptide-1 (GLP-1) is an incretin hormone that induces glucose-dependent stimulation of insulin and reduction in glucagon secretion, delays gastric emptying and decreases appetite.^1^,^2^ Consequently, there has been much interest in GLP-1 as a treatment for type 2 diabetes. However, its therapeutic application is limited by its rapid degradation by dipeptidyl peptidase-4 (DPP-4),^3^ resulting in a half-life of approximately 1 h after subcutaneous administration.^4^

Liraglutide is a human GLP-1 analogue designed to provide a longer duration of action. The peptide is linked via a γ-l-glutamyl spacer to a 16-carbon fatty acid residue.^5^ This facilitates reversible self-association and binding to serum albumin, slowing release from the injection site and reducing degradation by DPP-4, resulting in a plasma half-life of approximately 13 h.^6^ Its apparent volume of distribution is low (11–17 L),^6^ approximating the distribution volume of albumin, which is almost identical to the total volume of blood and interstitial fluid (∼15 L),^7^ thus indicating high levels of plasma protein binding.

Plasma protein binding of a drug can be altered by many factors, including disease state, age and concomitant therapies. Hepatic impairment and nephrotic syndrome, both of which are common among patients with type 2 diabetes and the elderly, are associated with hypoalbuminaemia,^8^ which could potentially lead to altered protein binding levels of a drug. Plasma protein binding of a drug is clinically important, as it can affect the predictability of its pharmacokinetics, pharmacodynamics and dose–response relationship.^9^ Plasma protein binding is critical to the protracted duration of action of liraglutide; therefore, it is important to determine whether any of these conditions alter liraglutide plasma protein binding.

Standard methods for determining drug binding to plasma proteins include: *in vivo* measurements, gel filtration, ultracentrifugation, ultrafiltration and equilibrium dialysis. Ultrafiltration is the method of choice for drugs that are not quickly degraded or metabolised.^10^ However, it has previously been observed that lipophilic drugs, such as liraglutide, insulin detemir and other drugs with 16-carbon fatty acid moieties, adsorb to the filter membrane rather than passing through it.^11^ Initial attempts to quantify plasma protein binding using standard equilibrium dialysis were also hindered by adsorption to the dialysis membrane. To overcome this problem, we developed a hitherto unpublished reiterated stepwise equilibrium dialysis method, which has been utilised to measure the plasma protein binding of insulin detemir.^11^

This methodology was later optimised to quantify liraglutide plasma protein binding.^12^ Here, we describe the reiterated stepwise equilibrium dialysis method, its validation and use to determine the extent of *in vitro* binding of liraglutide to plasma proteins.

## MATERIALS AND METHODS

All studies were performed according to good laboratory practice, and all reports formed part of the documentation submitted to the health authorities for the approval of liraglutide.

### Materials

Plasma was derived from ethylenediaminetetraacetic-acid-treated human blood, obtained under fasting conditions from two male and two female healthy volunteers who provided written informed consent. Liraglutide [molecular weight (MW) 3.751 kg/mol] was obtained from Novo Nordisk A/S (Bagsvaerd, Copenhagen, Denmark). Human serum albumin (HSA) and α1-acid glycoprotein (AAGP) were obtained from Sigma–Aldrich (Gillingham, UK).

### Methods

#### Radiolabelling of Liraglutide and Stock Solution Preparation

Liraglutide was radiolabelled to allow quantification by liquid scintillation counting. [^125/127^I Tyr 19]-labelled liraglutide was prepared and purified at the Chemistry and Isotope Laboratory, Novo Nordisk A/S, on the day before the dialysis incubation. Labelling was performed by the lactoperoxidase/hydrogen peroxide method, followed by purification and radiochemical purity analysis by high-pressure liquid chromatography as previously described.^13^ The radiochemical stability of [^125/127^I Tyr 19]-labelled liraglutide was improved by diluting the Na[^125^I] 20-fold with Na[^127^I] before iodination. This produced a tracer with low specific activity (0.1 μCi/pmol) and high radiochemical purity (>99%), which was maintained after 1 month at −20°C.

Liraglutide stock solution (10^7^ pM) comprised 3.8 mg liraglutide and 40 μL iodinated liraglutide (corresponding to 10 μCi/mL) in 100 mL Krebs–Henseleit (KH) buffer (pH 7.4).^14^ Different liraglutide concentrations were obtained (10^5^ and 10^3^ pM) by serial dilution, as appropriate. Final liraglutide concentrations of 10^6^, 10^4^ and 10^2^ pM were obtained by 1:10 dilution into plasma. A plasma-free buffer incubation solution was also prepared.

#### Reiterated Stepwise Equilibrium Dialysis Assay

**Figure 1:**
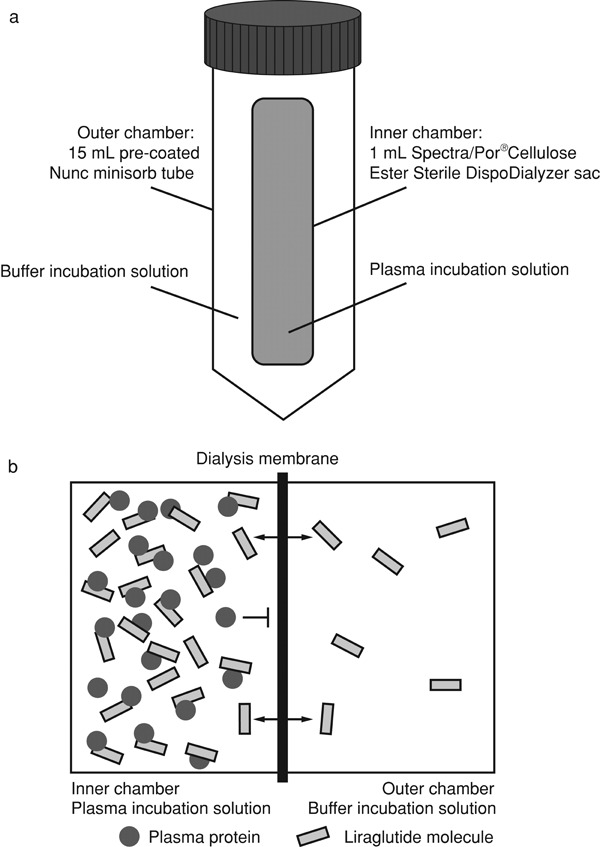
The reiterated stepwise equilibrium dialysis assay. (a) Illustration of the experimental set-up used to perform the assay; (b) schematic representation of the inner and outer chamber buffer solutions at the start of the assay.

A standard equilibrium dialysis experimental set-up is illustrated. Figures 1a and 1b show the modified assay set-up, which was initiated with liraglutide in both chambers to avoid it becoming trapped in the dialysis membrane. Experiments were repeated at different across-membrane liraglutide concentration ratios (inner–outer) until liraglutide passage through the dialysis membrane was minimal, indicating that the system was at equilibrium.

The experimental set-up consisted of a Spectra/Por® Cellulose Ester Sterile DispoDialyzer sac (Spectrum Europe B.V., Breda, The Netherlands) containing 1.0 mL plasma (or HSA/AAGP) incubation solution with the appropriate concentration of liraglutide, dependent on the across-membrane concentration ratio required. Dialysis sacs had a diameter of 5 mm, a declared inner volume of 1 mL (although only an effective volume of 0.7 mL can be drawn) and a MW cut-off of 15 kDa, which provided the optimal balance between robustness and passage of the molecule. Dialysis sacs were mounted in an outer chamber filled with 12.5 mL buffer incubation solution, which contained the appropriate concentration of liraglutide in KH buffer. The outer chamber consisted of a screw-capped Minisorb tube (catalogue #366060; Nunc, Roskilde, Denmark), with an effective volume of 15 mL. The outer chamber was pre-coated with liraglutide by incubating twice with buffer incubation solution for 30 min at 37 ± 2°C in a Memmert thermostatic incubator (Memmert GmbH & Company KG, Büchenbach, Germany), followed by a final overnight incubation. Forty-five millilitre of buffer incubation solution (sufficient buffer for triplicate incubations) was prepared in 50 mL screw-capped Minisorb tubes (PBC catalogue #339497; Nunc).

Following pre-coating, dialysis sacs were mounted into the outer buffer chambers and incubated at 37 ± 2°C in a Memmert thermostatic incubator for 4–5 h with mixing using an IKA-Schuttler MTS 2 electronic mixer (IKA®-Werke GmbH & Company KG, Staufen, Germany) at the lowest step of horizontal rotation. Experiments were performed in triplicate.

#### Sampling, Counting and Calculation of Binding Levels

After incubation, dialysis sacs were visually inspected for yellow colouring of the outer buffer solution, indicating invisible leakage. Extremely high radioactivity counts were also the possible indicators of leakage.

Samples (0.7 mL) were extracted from inside the dialysis sac, and 1 mL was extracted from the outer chamber. Samples were counted for 10 min in a Wallac 1470 Wizard gamma counter (PerkinElmer, Waltham, Massachusetts).

The percentage of the bound drug at equilibrium, bound (%) was calculated as follows:


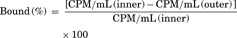


where CPM is the counts per minute.

Results were calculated as the percentage of the bound drug at equilibrium of concentration at pre-incubation. Mean (standard deviation, SD) percentage of bound liraglutide was calculated from each set of triplicate incubations using Excel (Microsoft™, Seattle, Washington); results were rejected if the SD was greater than 1.0.

#### Reproducibility Testing

Reproducibility was confirmed by assessing intra- and inter-day variation. To confirm stability of liraglutide, a quantitative recovery was performed using a liraglutide-specific enzyme-linked immunosorbent assay (ELISA) (Capio Diagnostik A/S, Copenhagen, Denmark) as described previously.^15^ Reproducibility assays were performed using undiluted plasma pooled from all four volunteers and the plasma incubation solution contained 10^4^ pM liraglutide. Experiments were performed at a liraglutide concentration ratio of 100:1 (inner–outer). Intra-day variation was evaluated in six assay runs performed on the same day, whereas inter-day variation was assessed by assays performed on 5 separate days.

#### Liraglutide Plasma Protein Binding

The experiment was repeated with plasma incubation solutions containing 10^6^, 10^4^ (representing a clinically relevant concentration)^6^ and 10^2^ pM liraglutide. Across-membrane inner–outer liraglutide ratios of 100:1 and 50:1 (corresponding to 99% and 98% liraglutide bound to plasma proteins, respectively) were investigated, as studies had previously shown close to 99% binding in human plasma.^12^ The effect of diluting the plasma sample to 50%, 10% and 5% was investigated at 10^4^ pM liraglutide, and at both 100:1 and 50:1 inner–outer liraglutide ratios.

#### Liraglutide Binding to HSA and AAGP

The HSA incubation solution contained approximately 7.4 × 10^−4^ M (50 mg/mL) HSA (representing the physiological concentration in plasma)^16^ and 10^4^pM liraglutide, diluted in KH buffer. Inner–outer liraglutide ratios of 100:1 and 50:1 were used to investigate HSA binding (corresponding to 99% and 98% bound, respectively).

α1-acid glycoprotein incubation solution contained approximately 2.4 × 10^−5^ M (1 mg/mL) AAGP (representing the physiological concentration in plasma)^17^ and 10^4^ pM liraglutide, diluted in KH buffer. AAGP binding was assessed at liraglutide inner–outer ratios of 2:1, 5:1, 10:1, 20:1, 50:1 and 100:1, corresponding to 50%, 80%, 90%, 95%, 98% and 99%, respectively, on the presumption that binding would be approximately 50% because of the results observed with other acylated polypeptides (Plum, unpublished data).

#### Statistical Analyses

The arithmetic mean (SD) was calculated for each concentration level and analysed using a two-sided analysis of variance (ANOVA) for gender and concentration effect. ANOVAs were performed using SAS version 9.3 (SAS Institute Inc., Cary, North Carolina).

## RESULTS

### Reproducibility Testing

To confirm reproducibility, intra- and inter-day assay evaluations were performed (Table 1). The intra-day coefficient of variation (CV) was 0.10%, and the inter-day CV was 0.11%, demonstrating good reproducibility. The mean (SD) concentration of liraglutide recovered was 5883 (668) pM, as analysed by ELISA, corresponding to 59% of the target concentration.

**Table 1:** Intra- and Inter-Day Variation in Liraglutide Protein Binding

Intra-Day	Mean (SD) Liraglutide Bound [%]	Inter-Day	Mean (SD) Liraglutide Bound [%]
Run 1	99.9 (0.03)	Day 1	99.9 (0.03)
Run 2	99.7 (0.35)	Day 2	99.8 (0.04)
Run 3	99.8 (0.06)	Day 3	99.8 (0.04)
Run 4	99.9 (0.03)	Day 4	99.8 (0.02)
Run 5	99.7 (0.22)	Day 5	99.6 (0.19)
Run 6	99.8 (0.05)		
Overall mean (SD)	99.8 (0.10)		99.8 (0.11)
Coefficient of variation (%)	0.10		0.11

Data are unadjusted means (SD).

Liraglutide concentration of the plasma incubation solution was 10^4^ pM, and the inner–outer liraglutide ratio was 100:1.

The intra-day evaluation involved repetition of the assay six times in 1 day, whereas the inter-day assay involved repetition of the assay on 5 separate days.

### Liraglutide Plasma Protein Binding

The assay was used to determine the percentage of circulating liraglutide that was bound to plasma proteins in a previously published study.^11^ Results from four healthy individuals are shown in Table 2. At a clinically relevant concentration of 10^4^ pM,^6^ 98.7%–99.2% liraglutide was bound to protein.^11^ Dilution to 10^2^ pM did not significantly affect protein binding compared with 10^4^ pM (estimated difference 0.05%; *p* = 0.581). When the concentration was increased to 10^6^ pM, protein binding was significantly reduced compared with 10^4^ pM (estimated difference: −0.33%; *p* = 0.009) and 10^2^ pM (estimated difference: −0.38%; *p* = 0.0047). There was no significant effect of gender on the proportion of protein-bound liraglutide (estimated difference females vs. males: −0.18%; *p* = 0.593). Furthermore, dilution of the plasma (50% to 5%) did not affect the extent of liraglutide plasma protein binding (Table 3).

**Table 2:** Extent of Liraglutide Protein Binding in Plasma Collected from Healthy Volunteers

	Mean (SD) Liraglutide Bound [%]				
Liraglutide Concentration of the Plasma Incubation Buffer (pM)	Male 1	Male 2	Female 1	Female 2
Inner–outer ratio (100:1)				
10^6^	99.0 (0.5)	98.6 (0.3)	99.0 (0.2)	98.3 (0.4)
10^4^	99.2 (0.5)	99.2 (0.5)	99.1 (0.3)	98.7 (0.6)
10^2^	99.3 (0.3)	99.0 (0.5)	99.3 (0.5)	98.8 (0.4)
Inner–outer ratio (50:1)				
10^6^	98.4 (0.9)	97.6 (0.5)	98.6 (0.3)	97.3 (0.3)
10^4^	99.4 (0.1)	98.2 (0.2)	98.9 (0.8)	97.0 (0.8)
10^2^	99.2 (0.2)	99.1 (0.2)	99.2 (0.1)	97.9 (1.0)

Data are unadjusted means (SD)

**Table 3:** Effect of Plasma Dilution on Percentage of Liraglutide Bound to Plasma Proteins

	Mean (SD) Liraglutide Bound [%]		
Plasma (%)	Inner–Outer Ratio (50:1)	Inner–Outer Ratio (100:1)
100	99.4 (0.1)	99.2 (0.5)
50	98.8 (0.5)	99.3 (0.3)
10	99.2 (0.1)	99.2 (0.5)
5	99.3 (0.0)	99.5 (0.3)

Data are unadjusted means (SD). Liraglutide concentration of the plasma incubation buffer was 10^4^ pM.

### Liraglutide Binding to HSA and AAGP

Human serum albumin is the most abundant protein in human plasma,^18^ whereas AAGP is a plasma glycoprotein that is elevated in some disease states.^19^ In order to quantify HSA and AAGP binding of liraglutide, the assay was performed using physiological concentrations of HSA and AAGP (50 and 1 mg/mL, respectively).^17^ At a liraglutide concentration of 10^4^ pM, and inner–outer ratio of 100:1, more than 99% of liraglutide was bound to HSA and AAGP (Table 4).

**Table 4:** Liraglutide Binding to Human Serum Albumin and α1-acid Glycoprotein Across a Range of Inner–Outer Liraglutide Ratios

	Mean (SD) Liraglutide Bound [%]		
Inner–Outer Ratio (%)	HSA	AAGP
1:1 (50)	n/p	55.7 (3.8)
5:1 (80)	n/p	83.8 (1.0)
10:1 (90)	n/p	93.0 (0.1)
20:1 (95)	n/p	96.9 (0.1)
50:1 (98)	99.0 (0.0)	98.7 (0.1)
100:1 (99)	99.4 (0.1)	99.3 (0.0)

Data are unadjusted means (SD). Liraglutide concentration of the plasma incubation buffer was 10^4^ pM.

n/p, not performed.

## DISCUSSION

The present study describes a novel assay that can be used to quantify the proportion of liraglutide bound to plasma proteins *in vitro*. It was used to determine a previously reported finding^12^ that at a clinically relevant liraglutide concentration of 10^4^ pM,^6^ approximately 99% of liraglutide was bound to plasma proteins. Protein binding was significantly reduced at a higher concentration of liraglutide (10^6^ pM), but the absolute difference was small (0.4%) and unlikely to be clinically relevant, given that 10^6^ pM is approximately 50-fold higher than steady-state levels in humans. Gender had no apparent effect on binding, although this should be interpreted with caution given the small sample size. The extent of liraglutide protein binding was not affected by dilution of the plasma sample to 5% concentration. Liraglutide was highly bound to both HSA and AAGP.

As with many plasma-protein-bound drugs, only the unbound fraction of liraglutide interacts with the effector to produce a pharmacological response.^19^ This assay showed that 98%–99% of liraglutide was bound to plasma proteins.^12^ Therefore, at a clinically relevant liraglutide concentration (10^4^ pM), the unbound liraglutide concentration would be 100–200 pM, which is in accordance with the half maximal effective concentration value for liraglutide.^5^ If the extent of plasma protein binding of a drug becomes altered, then it can affect the volume of distribution and rate of clearance.^9^

The effect of reduced plasma protein levels on liraglutide protein binding is of clinical relevance. Hepatic impairment is common among people with type 2 diabetes and often results in reduced levels of HSA. In a pharmacokinetic study of liraglutide in patients with hepatic impairment, the lowest level of HSA was 3 × 10^−4^ M (∼20 mg/mL),^12^ 20-fold higher than that in the 5% plasma solution [∼1.5 × 10^−5^ M (1 mg/mL)] analysed herein. This indicates that hepatic impairment is unlikely to reduce liraglutide plasma protein binding. Furthermore, physiological levels of HSA and AAGP (∼10^−3^ and 10^−5^ M, respectively) are 1000- to 10,000-fold higher than clinically relevant concentrations of liraglutide; therefore, the reduced plasma protein levels observed in hepatic impairment are unlikely to affect liraglutide binding. Indeed, in the pharmacokinetic study of Flint et al.^12^, the unbound fraction of liraglutide was very low in all groups, with no clear association with hepatic impairment. At a liraglutide concentration of 10^2^ pM, mean (SD) unbound percentages were 0.53% (0.46) in healthy subjects, as compared with 0.59% (0.61), 0.68% (0.45) and 0.40% (0.2) in those with mild, moderate and severe hepatic impairment, respectively.^12^

Renal impairment is another common condition associated with type 2 diabetes that can result in marked hypoalbuminaemia.^8^ Reassuringly, liraglutide pharmacokinetics was not altered in a study of subjects with mild-to-severe renal impairment versus healthy subjects.^20^

The lipophilic nature of 16-carbon fatty acylated peptides such as liraglutide can make it difficult to quantify plasma protein binding by ultrafiltration, as they can become trapped within the filter membrane.^11^ The methodology reported herein represents a novel approach to quantify liraglutide protein binding *in vitro*. Inclusion of liraglutide in the plasma/protein incubation solution, as well as in the buffer incubation solution, prevented the lipophilic molecule from becoming trapped in the dialysis membrane. Furthermore, pre-coating of the chamber with liraglutide prevented the loss of the drug through adsorption to the surface of the chamber. Together, these modifications allowed the measurement of liraglutide binding to plasma proteins with good reproducibility. The dialysis sac volume used in this assay is relatively large (1 mL); therefore, when considering the small volumes of plasma that are normally obtained from patient populations, the ability to dilute samples to 5% in this assay was advantageous. However, dilution should be performed with reference to the albumin status of patients and the plasma concentrations of the drug.

The high level of plasma protein binding demonstrated in this study is part of the mechanism through which liraglutide's plasma half-life is extended. Its prolonged duration of action ensures that constant exposure is achieved with once-daily dosing. As type 2 diabetes patients have a blunted GLP-1 response, it is likely that the constant exposure of liraglutide is important for its efficacy. The GLP-1 receptor agonist exenatide does not demonstrate significant levels of plasma protein binding and has a shorter duration of action, necessitating twice-daily administration.^21^ This may help to explain the greater glycaemic efficacy achieved with liraglutide compared with exenatide.

## CONCLUSIONS

The reiterated stepwise equilibrium dialysis assay described herein is suitable for the *in vitro* quantification of liraglutide protein binding. The assay has been used to quantify the high plasma protein binding levels of liraglutide (∼99%),^12^ a property that underlies its prolonged duration of action. The high physiological levels of plasma proteins relative to liraglutide indicate that its binding is unlikely to be affected in conditions associated with lower albumin levels, such as hepatic impairment. In the future, this assay could be used to determine the effect of other highly plasma-protein-bound drugs on liraglutide plasma protein binding. The methodology could also have applications in determining the plasma protein binding of other lipophilic drug molecules that are unsuitable for ultrafiltration.
